# Nurse-led medication management as a critical component of transitional care for preventing drug-related problems

**DOI:** 10.1007/s40520-024-02799-3

**Published:** 2024-07-26

**Authors:** Yingting Han, Jia Chen, Yulei Xu, Peihua Huang, Lili Hou

**Affiliations:** 1 Department of Nursing, Renhe Hospital, Baoshan District, No. 1999, West Changjiang Rd, Baoshan District, Shanghai, 200431 China; 2 Department of Nursing, Ninth People’s Hospital, Shanghai Jiaotong University School of Medicine, No. 639, Zhizaoju Rd, Huangpu District, Shanghai, 200011 China

**Keywords:** Transitional care, Drug-related problems, Medication reconciliation, Nurse-led intervention

## Abstract

Drug-related problems (DRPs) are critical medical issues during transition from hospital to home with high prevalence. The application of a variety of interventional strategies as part of the transitional care has been studied for preventing DRPs. However, it remains challenging for minimizing DRPs in patients, especially in older adults and those with high risk of medication discrepancies after hospital discharge. In this narrative review, we demonstrated that age, specific medications and polypharmacy, as well as some patient-related and system-related factors all contribute to a higher prevalence of transitional DPRs, most of which could be largely prevented by enhancing nurse-led multidisciplinary medication reconciliation. Nurses’ contributions during transitional period for preventing DRPs include information collection and evaluation, communication and education, enhancement of medication adherence, as well as coordination among healthcare professionals. We concluded that nurse-led strategies for medication management can be implemented to prevent or solve DRPs during the high-risk transitional period, and subsequently improve patients’ satisfaction and health-related outcomes, prevent the unnecessary loss and waste of medical expenditure and resources, and increase the efficiency of the multidisciplinary teamwork during transitional care.

## Introduction

Transitional care refers to ‘a set of actions designed to ensure the coordination and continuity of patient care and to prevent poor outcomes when patients transfer to different locations or different levels of care within the same location’ [1–4]. An optimal transitional care requires a multidisciplinary teamwork and may involve participation and lifestyle modifications for patients and their caregivers [5]. For older adults, medication management is one of the most important and challenging components for reducing length of hospital stay, readmissions, healthcare cost and mortality [6–9], since medication regimens could be significantly altered during hospitalization due to acute medical conditions or new diagnoses. Notably, medication discrepancies (defined as any difference between discharge medication list and medications patients report actually taking post discharge) were observed in more than half (56%) of the elderly patients during the first 48 h post-discharge in cross-sectoral transitional period [10], which may lead to medication errors (defined as failures in the treatment process that (potentially) lead to harm to the patients) [11]. As a matter of fact, nearly half (49%) of the patients have been reported to experience at least one medication error in continuity of care [12]. A successful medication management is not only essential for preventing/terminating medication discrepancies or errors, but also critical for reducing drug waste. Nurses play an important role in medication management during transitional period. The aim of this narrative review was to summarize from existing research the contributing factors for post-discharge drug-related problems (DRPs) and the nurses’ role in transitional care for preventing DRPs, as well as to explore the nurse-led interventional strategies in the hope of supporting them as liaison officers in this multidisciplinary teamwork.

## Literature search strategy

A literature search was performed on PubMed database using keywords ‘transitional care’, ‘medication reconciliation’, ‘drug-related problem’. Specifically, the search strategy was (transitional care) AND ((drug-related problem) OR (medication reconciliation)). A total of 113 English articles on human studies were collected for subsequent screening. Titles and abstracts of these articles were assessed by two authors independently, and those relevant to the searched terms were processed for further detailed review. For any discrepancies occurred, they were evaluated by a third author. Additional articles were selected when applicable based on articles in these searches and selections. Irrelevant topics, reviews, systematic reviews and/or meta-analyses, case reports and case series have all been excluded.

## DRPs and their contributing factors

**Fig. 1:**
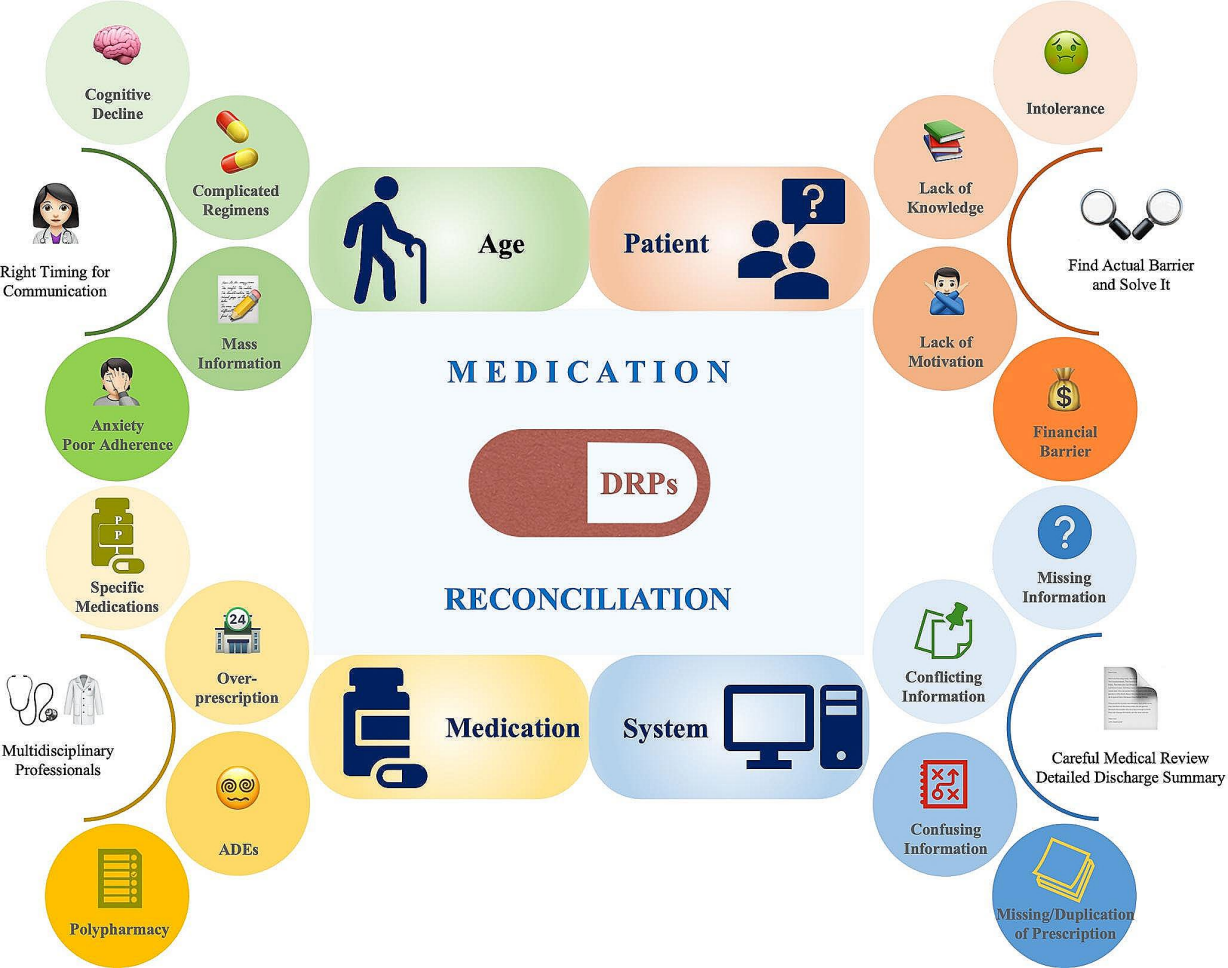
Common contributing factors of drug-related problems (DRPs) and potential solutions

DRPs, as defined by the Pharmaceutical Care Network Europe (PCNE), are a group of events or circumstances involving drug therapy that actually or potentially interferes with desired health outcomes (Fig. 1). According to previous studies, the majority of patients (84%) were observed to be affected by at least one DRPs in geriatric rehabilitation centers [13], whereas an average prevalence of 81% and even a higher occurrence of 94.4% were detected in acute hospitals [14] and in long-term care hospitals [15], respectively. Among the subcategories of DRPs, the drug-drug interaction (34.6%) was the most pronounced one analyzed by a university hospital [16], whereas non-adherence (18%) was the most common one identified by pharmacists from an academic family medicine outpatient clinic [17]. The percentages of identified DRPs occurred before hospital admission, on the ward and during transitional care were 37%, 36% and 27%, respectively [16]. DRPs occurring during transitional period may lead to adverse outcomes ranging from patients’ discomfort or dissatisfaction to adverse drug events (ADEs) [18], which subsequently result in worse prognosis, prolonged hospital stay, elevated occurrence of hospital readmissions, and increased utilization of healthcare resources [19, 20].

### Age

Notably, older adults are particularly vulnerable to DRPs following discharge due to multiple factors [21, 22], including chronic comorbid medical conditions, metabolic changes, functional and cognitive impairments, complicated therapeutic regimens administered (often with prescriptions from several medical providers), and extensive changes in their drugs during hospitalization. In a meta-analysis, it displayed that older adults had a three-fold and a seven-fold increase in prevalence of ADEs compared to adult and pediatric patients, respectively [23]. DRPs in older adults may result in frequent readmissions [22], especially during hospital-to-home transitional period, as various information is provided at this stressful time, which makes it difficult for patients and their caregivers to understand and remember [3, 24]. Subsequently at home, the uncertainty of new drugs from the elderly patients may lead to their anxiety and poor adherence to treatment. Therefore, it was critical to find a right timing to provide information on medication management to patients and caregivers at or soon after discharge.

### Specific drugs and polypharmacy

As shown by previous studies, drugs that were prescribed more often accounted for approximately half of the medication discrepancies. Specifically for several classes of drugs, anticoagulants accounted for 13%, diuretics accounted for 10%, angiotensin-converting enzyme inhibitors (ACEIs) accounted for 10%, and proton pump inhibitors (PPIs) accounted for 7% of medication discrepancies generated during transitional care [17]. Moreover, in a prospective analysis of 3695 patient-episodes, it showed that patients experiencing ADRs were more likely to take diuretics, opioid analgesics and anticoagulants, leading to a longer length of hospital stay and a higher financial cost to both patients and healthcare systems [25]. Therefore, these classes of drugs are considered as frequent ones for DRPs during both hospitalization and post-discharge transition. The problem of overprescribing PPIs has been reported in multiple studies [26, 27], which could be significantly prevented by transitional interventions [13] for protecting patients from long-term negative effects such as enteric infections.

In addition, polypharmacy is also regarded as a contributing factor for DRPs. For instance, patients with chronic kidney disease (CKD) often take an average of 6 to 12 medications, and the incidence of polypharmacy was approximately 80% in this cohort [28]. Therefore, polypharmacy and/or high-risk drugs (e.g., anticoagulants) are considered as high-risk factors for post-discharge DRPs. The risk of ADEs was increased by 13% when two drugs are used together, by 58% when using five drugs, and reached as high as 82% when using seven or more drugs simultaneously [29]. Such risks of ADEs could be reduced by transitional care interventions utilizing multicomponent approaches and reconciled medications [30].

### Patient-related factors

Patient-related factors often include adverse drug reactions (ADRs), intolerance, lack of knowledge about drugs, lack of motivation in taking drugs, as well as financial barriers [31]. Among these factors, 70% were intentional non-adherence, leading to the discrepancies between registered prescriptions and patients’ actual use of drugs [32]. Thus, it is critical for medical professionals to identify each individual’s actual barriers at an earliest timepoint and find out a solution to each barrier to avoid intentional non-adherence.

### System-related factors

In a study of participants at the age of 50 + years, the most common contributing factor for system-level medication discrepancies was incomplete or inaccurate discharge instructions (also known as error of omission) [13, 33], which are yet modifiable by careful medical reviews for optimizing in-hospital drugs and for standardizing discharge summaries and medication lists. System-level discrepancies may also occur during the long-term medical care after acute discharge, as noticed in a recent study in Denmark that Shared Medication Record (SMR) was not updated regularly by GPs and information on drug changes was missing after discharge or at referral [34]. Meanwhile, DRPs in SMR including dispensing errors at hospital and missing electronic prescriptions have also been found [34]. In one previous study, it showed that nurses identified more system-level discrepancies (in 69% of participants) than patient-level discrepancies (in 40% of participants) during transitional period [33], whereas in other studies, a nearly equal proportion of these two types of discrepancies have been detected by nurses [17, 35]. Additionally, the presence of conflicting information from different sources or confusion between brand and generic names, which may confuse patients without sufficient guidance or education of relevant knowledge, is another frequent contributing factor for system-level medication discrepancies [33]. In the same study [33], duplication has also been pointed out as one of the most frequent contributing factors, which could be a consequence of (1) an inaccurate medication history collected at admission; (2) failure to reconcile home drugs recorded during admission with drugs shown on discharge summary; (3) ineffective or insufficient discharge education on drugs; (4) multiple prescribers who do not get access to patients’ accurate medication lists; (5) changes during formulary substitution. As studied previously, medication discrepancies identified on transition from a hospital to a skilled nursing facility observed that 19% were therapeutic duplications [36].

### Medication reconciliation

Medication reconciliation is a systematic process with a series of steps, which includes listing medications that are currently under and needed, comparing these lists and generating a new list, as well as communicating with patients and care providers subsequently on the new list [37]. The comparison of these medication lists may be performed at admission, transfer or discharge to avoid inconsistencies [38], which plays a critical part in reducing medication discrepancies and preventing DRPs during transitional care. Obtaining the best possible medication history has been revealed to be able to identify medication discrepancies [39]. A wide range of information sources could be acquired to meet the goal, with patients themselves and their caregivers as the main source of information collection. Other sources such as hospital medical records and shared electronic health records may also be utilized to enhance the accuracy of the collected history [40]. Apart from verification of medication history, medication reconciliation also involves in-depth assessment and clarification including reevaluation of drug’s indications and contraindications, appropriate doses for the patient and possible ADEs, by using the evaluator’s specific knowledge. Medication reconciliation also requires documentation of any changes made to the medication list to resolve discrepancies and optimize reconciled medication regimens. In addition, appropriate monitoring whilst taking the drugs, as well as sufficient laboratory tests for determining the continuation, dosage adjustment or termination of the drugs (e.g., as per renal or liver functions), are also parts of the medication reconciliation. It has been demonstrated that patients on Gastroenterology wards may benefit more from drug safety checks, whereas patients on Neurology wards may particularly benefit from drug-drug interaction checks, as compared to those in Urology department [16].

### Nurses’ role in transitional care for preventing DRPs

The handling of these DRPs was very time-consuming for healthcare professionals and costly for healthcare resources, therefore, it was widely recommended that more cross-sectoral interdisciplinary resources being put into the transitional care to prevent DRPs instead of solving the problems after they occur. After discharge, efficient communication and adequate follow-up is utmost necessary, since patients may confront adherence problems due to insufficient knowledge on drugs they take or inadequate understanding about their treatment or regimen complexity [41, 42]. Transitional care interventions vary in the cohort they target, the goal they set, the services and duration of support they provide, and the types of service providers. Various health professionals, such as clinical pharmacists and/or clinical pharmacologists, as well as physicians, may be involved in the prevention of DRPs during transitional period [43]. Here, we specifically focus on the nurse-led transitional care interventions targeting older adults in the prevention of DRPs (Table 1) to provide a profound thinking about the optimization of such interventions.

**Table 1:** Summary of RCTs of nurse-led post-discharge interventions during transitional care for preventing DRPs

Intervention (country)	Participants (age)	Primary Outcome	Secondary Outcome	Follow-up	Ref (publication year)
**Information collection and Evaluation**					
Home visit with the nurse case manager to facilitate discrepancy resolution for intervention group and usual care for control group (USA)	Patients ≥ 50 years of age (*n* = 220 in total, *n* = 110 for each group)	The percentage of medication discrepancies resolved ↑	The number of planned/emergent physician visits and readmission ↓ (trend)	8 weeks post enrollment	[35] 2009
Phone call f/u within 48–72 h p/d (USA)	Veterans (no age limit) (*n* = 730 in total, *n* = 605 for intervention period enrollees)	30-day readmission ↓ An estimated savings of $1225 per veteran net of programmatic costs	None	30 days p/d	[44] 2012
Phone call f/u within 48–72 h p/d (USA)	Patients ≥ 65 years of age from an academic hospital (*n* = 1247)	Medication discrepancies ↓ 30-day readmission ↓ An estimated net healthcare cost savings of $663 per person	None	30 days p/d	[45] 2016
Phone call f/u weekly for 30 days (beginning 24–72 h p/d) (USA)	Patients (no age limit) discharged from a rural hospital (*n* = 638 for intervention group, *n* = 2232 for control group)	Spending for Medicare fee-for-service beneficiaries ↓	14-day ambulatory care f/u rate (-), 30-day unplanned readmission rate (-), number of inpatient admissions (-), number of emergency department visits (-)	6 months p/d	[46] 2018
Phone call f/u using a scripted questionnaire for intervention group and a satisfaction survey for control group (USA)	Patients ≥ 65 years of age and discharged from ED (*n* = 2000)	30-day Return-to-the-ED/hospitalization/death (-)	None	30 days p/d	[47] 2018
Phone call f/u using a semi-scripted questionnaire for intervention group and a scripted satisfaction survey for control group within 24 h p/d (Netherlands)	Patients ≥ 70 years of age and discharged from ED (*n* = 4732 for intervention group, *n* = 5104 for control group)	30-day unplanned readmission/Return-to-the-ED (-)	None	30 days p/d	[48] 2021
**Communication and Education**					
Sepsis Transition And Recovery (STAR) Program (USA)	Patients (mean age 63.7 year) after sepsis hospitalization (*n* = 349 for intervention group, *n* = 342 for control group)	30-day mortality and readmission ↓	The number of days alive and outside the hospital (-), proportion with cause-specific rehospitalization (-), count of ED visits (-)	30 days p/d	[49] 2022
F/u visits by language-concordant study nurse to reinforce the care plan and to address acute complaints (USA)	Patients ≥ 55 years of age (*n* = 700)	HCAHPS nursing, medication and discharge communication domain scores (-); CTM-3 scores (-)	None	30 days p/d	[50] 2015
Nurse-led care versus GP-led care, involving individualized patient education and engagement and a treat-to-target strategy (UK)	Participants (no age limit) in the Nottingham Gout Treatment Trial phase II (*n* = 438)	Patient acceptability ↑, long-term adherence ↑, flare frequency ↑, gout knowledge	None	1 year after trial cessation	[51] 2020
A structured education program (China)	Patients ≥ 18 years of age with CHF (*n* = 96)	Medication adherence ↑, dietary modifications ↑, social support ↑, symptom control ↑	None	1 year after intervention	[52] 2019
A 6-week Nurse-led medication self-management intervention consisting of three one-on-one educational sessions on medication-related information, motivation and self-management skills (China)	Older patients ≥ 60 years of age with multimorbidity (*n* = 136 in total, *n* = 67 for intervention group, *n* = 69 for usual care group)	Medication adherence ↑	Medication self-management capacity ↑, treatment experience ↑, quality of life (-), utilization of healthcare services (-)	3 months p/d	[53] 2022
A 20-week Nurse-led training program consisting of health education and motivational meetings (Turkey)	Older patients (mean age 75.63 year) with hypertension residing in nursing homes (*n* = 74)	Blood pressure ↓, total cholesterol levels ↓, hypertension knowledge ↑, medication adherence ↑, quality of life ↑, BMI ↓, weight ↓, waist circumference ↓, hip circumference↓	None	24 weeks	[54] 2020
**Enhancement of Medication Adherence**					
A 12-week intervention consisted of regular text messages and phone calls (Turkey)	Patients at 40–64 years of age with primary hypertension (*n* = 92)	Medication adherence ↑; blood pressure control ↑	None	At the end of intervention	[55] 2022
A person-centered, nurse-led programme including 3 visits and 2 phone calls (Sweden)	Patients (mean age 71 year) with intermittent claudication and scheduled for revascularization (*n* = 214)	Adherence to prescribed secondary preventive medication (-)	Risk factors for CVD (-)	12 months after surgical treatment	[56] 2022
**Comprehensive intervention**					
(1) assessment of overall situation and comprehension, (2) discharge letter, (3) f/u phone call 2 days p/d (Denmark)	Non-surgical patients ≥ 18 years of age from acute medical unit (*n* = 200)	30-day readmission (-)	Utilization of healthcare (-); patient experience (-); HRQoL (-)	30 days p/d	[57] 2019
An integrated nurse-led tele-homecare program (China)	Patients > 65 years of age with chronic illnesses and high risk for readmission (*n* = 200)	The number of ED visits ↓; readmittance (-); mortality ↓	Patients’ medication adherence, activities of daily living, health status, QoL↑	6 months after intervention	[58] 2021
Semi-structured interviews (China)	Stroke survivors ≥ 18 years of age (*n* = 26) and caregivers (*n* = 33)	Self-care skills ↑	None	12 weeks	[59] 2023

↑ improved/increased; ↓ delayed/decreased; (-) no statistical significance

Abbr: f/u = follow-up; CHF = chronic heart failure; CTM-3 = Care Transitions Measure; CVD = cardiocerebrovascular disease; HCAHPS = Hospital Consumer Assessment of Healthcare Provider and Systems; HRQoL = health-related quality of life; p/d = post-discharge

### Information collection and evaluation

Documentation of identified discrepancies as well as resolutions of these discrepancies are of profound importance in the medication management during transitional period. It is a common responsibility for nurses to compare and contrast medication lists when a patient is transferred from one setting to another. To complete this task, nursing staff should gather all available medication lists, usually with discharge summary as the primary source. Nurses could assess the quality of discharge letter by using the following definition: a description of the active ingredient and an exemplary brand name, an explanation of drug changes in comparison to the home medication lists, a visualized presentation of the explanation next to each drug, and a recommendation for treatment duration of short-term drugs. Hohmann et al. [60] have conducted a clinical study on 312 patients with stroke or transient ischemic attack (TIA) who were randomized into intervention group (education + a detailed medication list) or control group (a brief discharge letter). Within a 3-month follow-up, the intervention group showed a higher rate (90.9%) of medication adherence as compared to that of the control group (83.3%) [60], indicating that the detailed medication list might be a good tool for information collection and evaluation. In addition, Meyer-Massetti et al. [61] have conducted a nurse-pharmacist collaboration study on 100 discharged patients receiving ≥ 4 medications, in which the quality of discharged medication prescriptions was effectively assessed using a PCNE Type 2b Medication Review by nurses.

Notably, patient interview is another main source of information to identify medication discrepancies, and these discrepancies should be incorporated into the final medication list. A home visit is recommended to be paid by a care coordinator within one week post discharge, since preventable ADEs often occur with the first 1–2 weeks post discharge [62, 63]. During the face-to-face visits or telephone calls, the interviewees should be asked to bring all the drugs (both prescribed and over-the-counter) to the visit or near the phone before questioning. Home visits are superior source of information to phone call visits for the following reasons. Firstly, home visits make patients feel more comfortable to share experiences and concerns about their drugs and more receptive to counselling due to face-to-face encounters, which were often used in nurse-pharmacist collaboration studies, either by integrating pharmacists into a home nursing service to identify DRPs [64], or reporting nurse-identified significant DRPs during home visits to community pharmacists [65]. Secondly, some specific risk factors, such as multiple storage locations and inappropriate storage conditions, can be more easily identified in patients’ own surroundings during home visits by nurses [66]. In addition, expired or spare drugs that are no longer prescribed should also be carefully checked to ensure that they are disposed of. The quantity of unused or expired drugs due to non-adherence, overprescribing, change of medical condition or therapeutic regimens could be directly obtained by pill counting or patient’s self-report. Thirdly, difficulties in memorizing or pronouncing drug names or accurately recalling dosage were observed in many participants in different studies, particularly for those with low health literacy and for older adults. The effectiveness in identifying and resolving medication discrepancies in patients during transitional period has been demonstrated by a pharmacist-nurse collaborated intervention [35]. As an alternative approach for post-discharge follow-up, telephone-based nurse-led models have shown some benefits in reducing 30-day rehospitalization rates from 34 to 23% [44] and in yielding estimated net healthcare cost savings of $663 per person [45]. Similarly, it was impressively reported that the post-discharge evaluation for care needs and medication reconciliation delivered by a primary care-based nurse care coordinator has successfully reduced post-discharge costs among 638 Medicare fee-for-service beneficiaries in the USA [46]. However, in two large-scaled RCTs on older patients and discharged from the emergency department (ED), a single scripted phone call from a trained nurse did not significantly alter the 30-day unplanned readmission rate, ED return or death [47, 48], indicating that phone call visits alone may be insufficient to produce good health-related outcome for patients discharged from acute conditions.

Furthermore, since duplication of therapy is one of the profound medication discrepancies affecting safety issues for older patients, it should be evaluated via listing drugs by their indications or therapeutic drug class [67]. Although it is frequently seen when a given indication is treated by more than one drug, it is rare to treat with multiple drugs from the same therapeutic drug class.

Several tools have been proposed and developed for further investigation of potential medication discrepancies. At hospital discharge, nursing staff may determine the Medication Regimen Complexity Index (MRCI) [68] based on the discharge prescription, and a higher MRCI represents a more complex regimen. At post-discharge visits, the Medication Discrepancy Tool (MDT) is a tool for identifying medication discrepancies and guiding resolutions particularly during transition between sites of care [33]; whereas the Comprehensive Medication Review (CMR) [69] could be used to evaluate ADRs (e.g., allergies), contraindications, treatment duplication, drug-drug or drug-disease interactions, dosages, treatment durations, abuse or misuse of drugs. Possible ADEs could be assessed by using a trigger list developed by Sino et al. [70] Standardized information collection using certain templates may improve the rates of high-quality documentation and reduce the rates of patients’ mortality within a year after discharge [71]. A structured drug report with an accurate list have been demonstrated in multiple studies to reduce the rate of medication errors and enhance patients’ adherence during the transitional care [72–74]. Recently, electronic systems and applications have been developed for the purpose of improving medication management during the transitional care. For instance, mHealth, an app developed for diabetic management, enabled users to view their recent prescriptions, record their daily administration of all drugs, and receive reminder to take drugs [75].

### Communication and education

Poor communication has been reported as one of the most important contributing factors for medication discrepancies and readmissions during transitional care [76]. Therefore, a comprehensive medication reconciliation strategy should include effective communication with the patients, their caregivers or responsible person in their institutional facilities, or both. It is frequently the nurses’ role to lead communications among patients/caregivers, hospitals, and the patients’ primary care providers during the transitional period. Direct communication is vitally important for collecting information that may require close monitoring and follow-up, which is a pivotal component when determining the plan of care. During the post-discharge contact, effective communication should enable patients and their caregivers to actively participate and express their beliefs, demands or concerns regarding their drugs [77], identify patient’s goal or preferences and address issues by asking if they have any questions [78]. This is particularly important since patients’ willingness to initiate and continue prescribed drugs is largely determined by their judgement on personal needs for the drug relative to their concerns about taking it [79]. Improved health-related outcomes as well as reduced healthcare costs have been observed when conducting interventions with patients’ goals as key motivators [80]. A combination of strategies emphasizing trust and plain language communications, as well as coordinating post-discharge activities such as medical referral and follow-up appointments may be used. It is necessary to instruct patients about the arrangement and importance of their follow-up appointments with clear verbal instructions. A positive communication involves explanations of the current situation, confirmation of the understanding of the situation and discussion about the future strategies. However, it is noteworthy that for older multi-lingual and cognitively impaired populations, higher-intensity interventions may be applied to improve discharge experience outcomes [50].

In addition, insufficient education and follow-up has been associated with reduced ability of patients to absorb information on drug-related information [81], which may cause misinformation or confusion and raise DRPs. In contrast, patients with a higher level of knowledge showed a better medication adherence and post-discharge behavior, indicating the essentiality of developing interventional programs that focus particularly on promoting patients’ knowledge. The lack of patients’ knowledge on drugs, which could be evaluated based on the study of Kwint et al. [82], may be resolved by follow-up visits during transitional period. It has been demonstrated that a 6-week nurse-led medication self-management intervention significantly improved medication adherence in an RCT on older individuals with multimorbidity [53], and a 20-week nurse-led training program consisting health education and motivational meetings improved medication adherence, health-related quality of life (HRQoL) and health-related indicators (e.g., blood pressure, cholesterol, body mass index (BMI), etc.) [54], suggesting nurses’ important role as a medication educator and motivator during transitional period. Appropriately conducted education with recall-promoting and teach-back techniques may allow for reiteration and refresh of important drug information, which has been shown to be effective in different settings [83]. The teach-back technique requires patients/caregivers to repeat their understanding of the drugs and teach the nurses who have provided the information during the education, otherwise, reeducation may be applied. To fulfill this task, nurses should be cognizant of drugs frequently implicated in discrepancies and be aware of drugs commonly used in the hospital that may not require long-term use. For instance, some drugs are prescribed PRN for pain or nausea should be discontinued on discharge despite frequency of dosing or patient-reported symptoms, which may result in uncontrolled pain or other side effects. Digital technologies such as mobile apps and multimedia have been increasingly employed in patient education. Previous studies have revealed that patients counselled via utilization of tablets were more likely to feel competent to make health decisions with their doctors, and were more willing to follow the doctors’ instructions [84]. In addition, individuals who used tablets for patient education gained more knowledge on self-injection of drugs compared with those receiving conventional explanations from nurses [85]. Furthermore, the self-management education with understandable and culturally-adapted materials was well acceptable and applicable among patients [86], resulting in higher self-efficacy [87].

### Enhancement of adherence

Typical medication adherence barriers for patients taking regular drugs for a long term are usually related to patients themselves, lack of appropriate care supports or medical resources, or system issues. Intentional nonadherence was the most common contributing factor for patient-level discrepancies. It was reported that only 78% of all electronic prescriptions and only 72% of new prescriptions were filled [88]. When digging out the reasons behind, most frequently, patients decided not to fill a prescription because they perceived the drugs were not necessary or helpful. Approximately 30% of the patient-level discrepancies were attributed to nonintentional nonadherence [33], which was due to patients not knowing that they were supposed to take a drug or a lack of knowledge about the dose and frequency of taking the drug as prescribed.

Firstly, delineation of patient’s actual drug taking behaviors is critical to identify the self-related adherence barriers. These behaviors include how often the patient misses a dose, whether there is a pattern to the dose missing and whether there is any difficulty administering or tolerating drugs. For instance, some drugs could be difficult to swallow or may cause stomach discomfort, while others (e.g., inhalers or injectables) may have complicated instructions for administration. There might be some other practical barriers for drug intake, e.g., forgetfulness, organizational problems or difficulties with opening pill containers, which can all be detected and solved by follow-up nurses. Notably, drug taking behaviors and adherence may sometimes be associated with individual’s beliefs and cultural background [79]. Occasionally, the patients may not be the best information providers, then interview with caregivers or examination of medical records from the facilities may provide a more accurate picture.

Secondly, enhancement of patient’s medication adherence is the main target with significant effectiveness in multiple nurse-led intervention studies [55]. The adherence could be evaluated and monitored by direct observation of drug consumption, symptomatic improvement, drug concentration in the blood or urine, pill counting and patient interview. Notably, adherence is possibly to be overestimated when using self-reported data compared with registry-reported observations [56]. To reinforce patients’ compliance with actual drugs, patients were taught in group classes (e.g., a fitness program/gym, smoking cessation program or healthy eating classes) regularly to establish lifestyle modification goals and develop personal action plans in the collaboration of responsible nurses [19].

## Coordination among healthcare professionals

Depending on the nurses’ scope of practice, his/her role may be as a collaborator to report identified medication discrepancies and DRPs to the patient’s multidisciplinary medical team (specialist, GP, pharmacist, nutritionist, physical therapists, etc.) [61, 66]. Coordination among healthcare professionals from different institutions by nurse coaches was detected as the power frame of the implementation of a health coaching program for stroke survivors and their caregivers [59]. Such coordination may facilitate tight linkages to patient’s specialists, other primary healthcare providers, as well as community care services for older adults and caregivers [89].

**Fig. 2:**
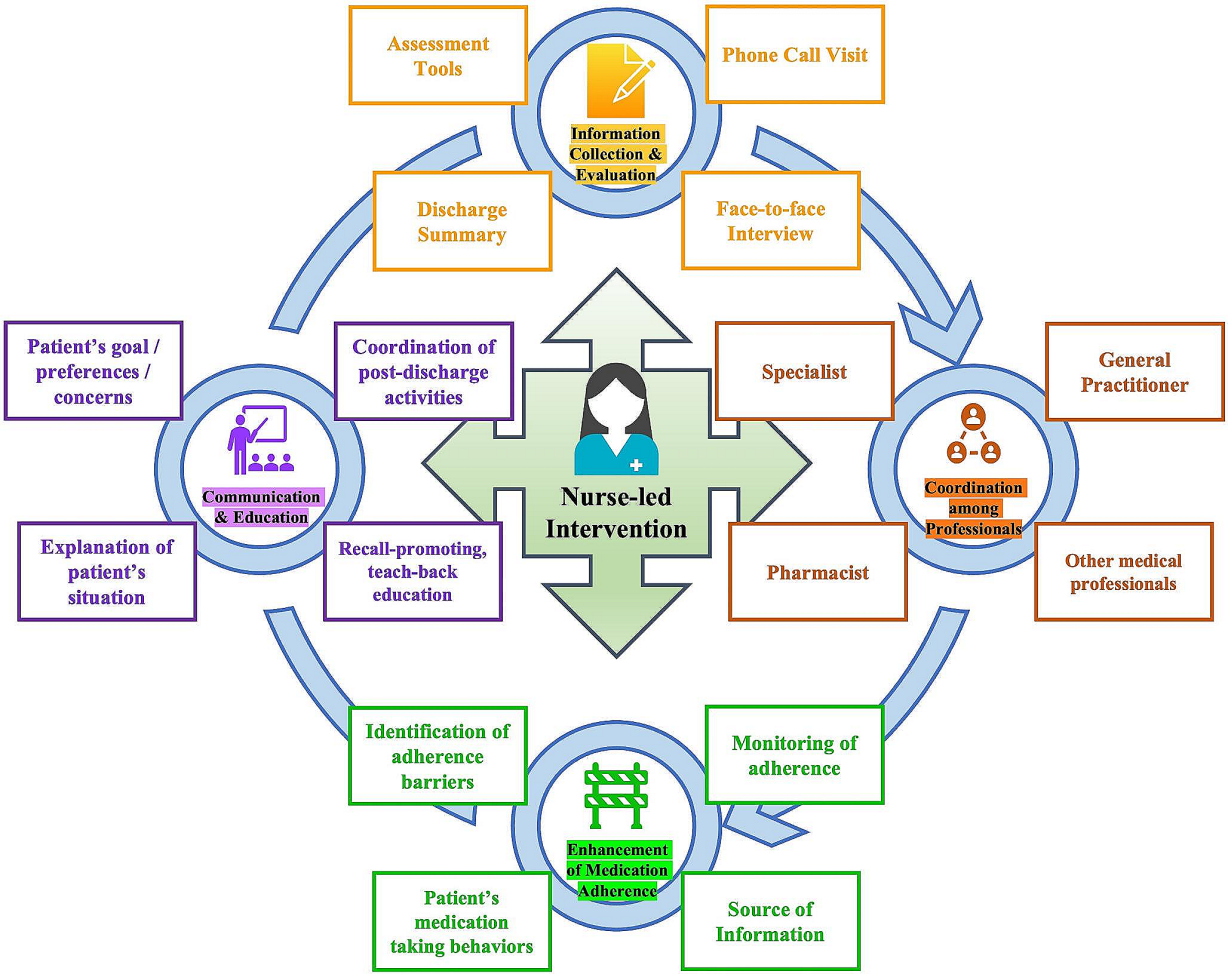
Nurses’ role in transitional care for preventing drug-related problems (DRPs)

Nurses are ideally positioned as ‘Liaison Officers’ who uniquely identify patients at high risk for DRPs and facilitates communications among different disciplines (specialists, pharmacists, primary care physicians, nursing home healthcare workers, etc.) and with patients and their families (Fig. 2). They are often the healthcare providers that contact most directly and frequently with patients and caregivers, and patients often feel more comfortable discussing symptoms and concerns with nurses, enabling earlier detection and report of potential side effects and ADRs, which may have not been identified by other healthcare professionals. Furthermore, nurses are frontline healthcare providers who are frequently involved in drug administration, providing them with a unique opportunity to monitor and record potential ADRs at an early stage [90]. A previously proposed evidence-based nurse-led transitional care model has been verified in multiple settings and medical providers to effectively improve the quality of care for individuals and families while reducing healthcare costs [91, 92], and the use of a transitional care bundle, which was delivered by nursing staff, pharmacy staff and physicians and developed to meet needs of patients, has reduced the medication errors in patients with a high risk of readmissions [93]. Nurses may play a crucial role in delivering this bundle as a ‘coordinator’ during transitional period. Specifically, at admission, how drugs had been taken by the patients would be discussed with nurses and physicians. In addition, education on discharge would also be provided by the nurses [93]. The main target of these interventions is to develop an evidence-based comprehensive and individualized plan of care to improve coordination of care and meet the needs and goals of patients and families, while more large-scaled RCTs are needed to test the effects of these nurse-led interventions.

## Conclusions

It has been shown that age, specific drugs and polypharmacy, as well as some patient- and system-related factors all contribute to a higher prevalence of transitional DRPs, most of which could be largely prevented by enhancing nurse-led multidisciplinary medication reconciliation. Nurses are ideally positioned as ‘Liaison Officers’ for preventing DRPs during transitional period, which includes information collection and evaluation, communication and education, enhancement of medication adherence, and coordination among healthcare professionals. Therefore, Nurse-led strategies for medication management can be implemented to prevent or solve DRPs during the high-risk transitional period, and subsequently improve patients’ satisfaction and health-related outcomes, prevent the unnecessary loss and waste of medical expenditure and resources, and increase the efficiency of the multidisciplinary teamwork during transitional care.

## Data Availability

No datasets were generated or analysed during the current study.
